# Comparison of different rapid screening tests and ELISA for HBV, HCV, and HIV among healthy blood donors and recipients at Jibla University Hospital Yemen

**DOI:** 10.25122/jml-2022-0051

**Published:** 2022-11

**Authors:** Abdullah Mohammed Al-Matary, Fadhl Ahmed Saed Al Gashaa

**Affiliations:** 1 Department of Human Medicine, College of Medical and Health Sciences, Jibla University, Jibla, Yemen; 2 Department of Biology, Al Farabi University College, Baghdad, Iraq; 3 Department of Medical Microbiology, College of Science, Ibb University, Ibb, Yemen

**Keywords:** HBV, HCV, HIV, ELISA, rapid diagnostic

## Abstract

Blood transfusion is associated with many risks, especially exposure to blood transfusion-transmitted infections considered one of the main causes of death worldwide, including hepatitis B (HBV) and C virus (HCV) and human immunodeficiency virus (HIV). The threat posed by blood-borne pathogens is disproportionately high, especially in developing countries, so there is a need for continuous monitoring of blood transfusions to prevent transmitting diseases. Rapid diagnostic immunochromatographic technique (ICT) methods are the most widely used methods in developing countries, although ELISA and molecular testing are considered more accurate worldwide. Therefore, the study aimed to compare the analytical sensitivity between rapid tests and the ELISA method for detecting HBV, HCV, and HIV infection among blood donors. Four hundred (400) blood donor samples were tested using the Rapid Test Kits (INTEC, SD, ABON, and CLUN), and the ELISA method was used as a confirmatory test. Out of 400 blood samples tested for viral infection, HBV, HCV, and HIV were detected in 8, 10, and 2 samples, respectively, using the ELISA technique. This study observed that the rate of sensitivity, specificity, positive predictive value (PPV), and negative predictive value (NPV), in addition to determining the diagnostic accuracy rate and error rate for all rapid diagnostic kits in detecting HBV, HCV and HIV are less accurate and associated with more false negatives compared to the ELISA technique. This study showed a significant difference in sensitivity between ELISA and rapid diagnostic immunochromatographic technique (ICT) groups; therefore, rapid diagnosis is not suitable for testing the quality of infectious markers for blood donors.

## INTRODUCTION

Blood transfusion services are an important part of the healthcare system, which is an important and essential life-saving treatment and part of the World Health Organization's list of essential medicines [1]. Worldwide, transfusion-transmitted infections (TTIs) remain a major health problem in most developing countries due to facilities with scarce resources and a shortage of staff members [2]. Bloodborne virus infections are most prominent in blood transfusion medicine. Sometimes blood donors carry an infectious agent without any signs and symptoms [3], and with each blood unit, there is a 1% chance of a related problem in transfusion-borne diseases [4].

Transfusion of infected blood to patients in need is a crime. Therefore, blood donors are screened for viral markers such as hepatitis B (HBV) and C virus (HCV), and human immunodeficiency virus (HIV) before transfusion to prevent transmission of infection. An important issue regarding blood safety is identifying infectious donors and preventing transmission to protect recipients. There are two important strategies for the success of blood transfusion. The first is the adaptation of the national blood transfusion policy to select donors, which aims to exclude donors with a high risk of infections such as HBV, HCV, and HIV. The second strategy is the application of methods with high specificity and sensitivity for identifying true positive and true negative individuals/blood units [5].

Different methods are used to diagnose viral infections, including enzyme-linked immunosorbent assay (ELISA), enzyme immunoassays (EIA), polymerase chain reaction (PCR), and immunochromatographic tests (ICT) [6]. Diagnostic methods used by ELISA, EIA, or PCR are costly, in addition to being used in advanced and well-equipped laboratories as well as major tertiary care hospitals [7]. Among all the techniques used for diagnosis, the ELISA technique is the preferred method of examination for the blood bank due to its effectiveness [8]. However, many blood banks still lack this technology and prefer rapid test kits because it is an easy-to-use, inexpensive method and does not require advanced equipment and detailed training [9].

Rapid tests are a rapid screening method used for the qualitative detection of infection in whole blood samples, serum, or plasma. Rapid tests use monoclonal and polyclonal antibodies to detect elevated levels of infection in samples [7]. ELISA is a type of “sandwich” enzyme immunoassay for detecting viral infections in plasma or serum. This test uses monoclonal antibodies due to its ability to bind to different subtypes of viral infections recognized by the World Health Organization (WHO) [10]. Rapid tests for viral infections have been available since the 1990s, intended for emergency diagnostics, home testing, and field surveys. In addition, rapid tests are being used to detect HBV, HCV, and HIV infections in many poor areas to overcome the lack of funding and equipment. However, a major concern with the use of rapid screening tests is that these tests must possess a high degree of sensitivity and an acceptable level of specificity to reduce false results [11].

The need for blood tests to detect HBV, HCV, and HIV in the serum of people in Yemen progressively increased primarily after the new rules of the Ministry of Health, which mandate these tests pre-operatively and during certain medical services like deliveries, blood transfusion, organ transplantations, traveling outside Yemen etc. The study was designed to investigate the sensitivity and specificity of four rapid tests (HBsAg, anti-HCV, anti-HIV), frequently used in different laboratories and hospitals in Yemen, and compare these with ELISA results. The aim of the study was also to recommend the most accurate and effective rapid tests for diagnosing HBV, HCV, and HIV in areas where advanced diagnostic facilities are not available.

## MATERIAL AND METHODS

A cross-sectional study was conducted on 400 healthy blood donors and recipients attending the Laboratories of Jiblah University Hospital, Yemen, between 2018 and 2019.

Four groups of the most common brands (INTEC, SD, ABON, and CLUN) used as rapid diagnostic kits for HBV, HCV, and HIV in different laboratories in Yemen were selected for the study. Moreover, the ELISA technique was used as a gold standard for comparative evaluation. In the Serology Laboratory of Jableh University, whole blood from participants was collected, and sera were separated and tested for HBV, HCV, and HIV viral markers using four different rapid diagnostic (ICT) groups and ELISA as a confirmatory diagnosis. The results of positive and negative samples performed with rapid tests for screening HBV, HCV, and HIV were compared with the results of confirmatory ELISA. Dual infections with HBV and HCV were excluded.

Data were analyzed using the Statistical Package for Social Sciences (SPSS) 11.0. Sensitivity, specificity, and positive and negative predictive values were calculated for all rapid test kits [12].

Sensitivity is the ability of a test to correctly identify patients with a disease and give a positive finding, expressed as a percentage.


$$\frac{\text{Person with disease detected positive by the screening test}}{\text{Total number of persons tested with disease}}\times 100$$

Specificity is the ability of a test to correctly identify people without the disease and give a negative finding, expressed as a percentage.


$$\frac{\text{Person without disease detected negative by the screening test}}{\text{Total number of persons tested without the disease}}\times 100$$

The positive predictive value is the proportion of cases receiving positive test results which are already patients. The negative predictive value is the proportion of cases receiving negative test results who are not patients yet.

## RESULTS

A total of 400 blood donors and recipients participated in the study. 75% (300) were males (most of them were donors), while 25% (100) were females (nearly all were recipients). There were 280 donors (70%) and 120 recipients (30%).

**Figure 1:**
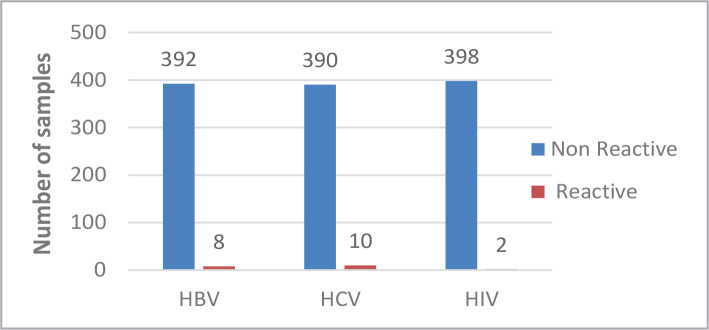
Prevalence of HBV, HCV, and HIV using the ELISA technique.

**Figure 2:**
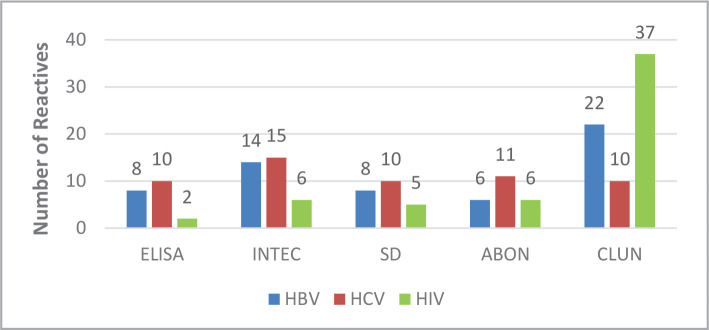
Positive results using ELISA and the four rapid tests.

**Figure 3:**
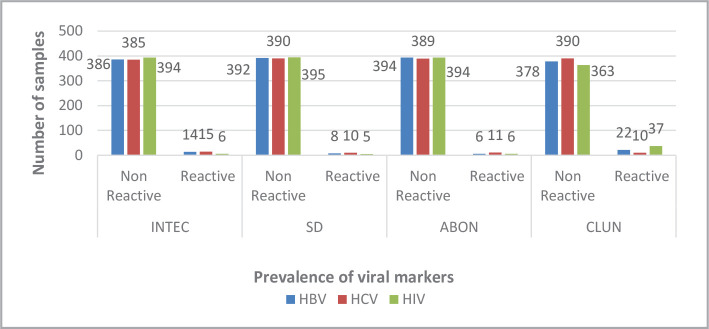
Prevalence of viral markers.

When the samples were tested using the ELISA method, there were 8 positive samples for HBV, 10 positive samples for HCV, and 2 for HIV (Figure 1). Nevertheless, when the samples were tested using a rapid method, there was a noticeable difference in sensitivity. Figures 2 and 3 show 14 (INTEC test), 8 (SD test), 6 (ABON test), and 22 (CLUN) samples positive for HBV. Furthermore, there were 15 (INTEC), 10 (SD), 11 (ABON), and 10 (CLUN) samples positive for HCV. Finally, 6 (INTEC), 5 (SD), 6 (ABON), and 37 (CLUN) samples were positive for HIV.

Tables 1, 2, and 3 show the rate of sensitivity, specificity, PPV, and NPV in addition to the rate of diagnostic accuracy and kappa statistic value (error rate) for all rapid diagnostic kits in detecting HBV, HCV, and HIV. These tests had less precision and were associated with more false negative results than the ELISA technique.

**Table 1:** Evaluation of rapid test kits for HBV compared with the ELISA technique.

Results of ELISA (n=400)		+ve						8					
		-ve						392					
Results for screening test (Kit for Hepatitis B) (n=400)													
Results of rapid test kits for Hepatitis B		Total	TP	TN	FP	FN	Prevalence (%)	Sensitivity (%)	Specificity (%)	PPV (%)	NPV (%)	Diagnostic accuracy (%)	Error rate compared to ELISA (%)
**INTEC**	+ve	14	6	384	8	2	3.2	75	98	42.9	99.5	97.5	2.5
	-ve	386											
**SD**	+ve	8	2	386	6	6	2	25	98.5	25	98.5	97	3
	-ve	392											
**ABON**	+ve	6	5	391	1	3	1.5	62.5	99.7	83.3	99.2	99	1
	-ve	394											
**CLUN**	+ve	22	6	376	16	2	5.5	75	95.9	27.3	99.5	95.5	4.5
	-ve	378											

**Table 2:** Evaluation of rapid test kits for HCV compared with the ELISA technique.

Results of ELISA (n=400)		+ve						10					
		-ve						390					
Results for screening test (kit for Hepatitis C) (n=400)													
Results of rapid test kits for Hepatitis C		Total	TP	TN	FP	FN	Prevalence (%)	Sensitivity (%)	Specificity (%)	PPV (%)	NPV (%)	Diagnostic accuracy (%)	Error rate compared to ELISA (%)
**INTEC**	+ve	15	8	383	7	2	3.8	80	98.2	53.3	99.5	97.75	2.25
	-ve	385											
**SD**	+ve	10	7	387	3	3	2	70	99.2	70	99.2	98.5	1.5
	-ve	390											
**ABON**	+ve	11	7	386	4	3	2.8	70	99	63.3	99.2	98.25	1.75
	-ve	389											
**CLUN**	+ve	10	6	386	4	4	25	60	99	60	99	98	2
	-ve	390											

**Table 3:** Evaluation of rapid test kits for HIV compared with the ELISA technique.

Results of ELISA (n=400)		+ve						2					
		-ve						398					
Results for screening test (kit for HIV) (n=400)													
Results of rapid test kits for HIV		Total	TP	TN	FP	FN	Prevalence (%)	Sensitivity (%)	Specificity (%)	PPV (%)	NPV (%)	Diagnostic accuracy (%)	Error rate compared to ELISA (%)
**INTEC**	+ve	6	2	394	4	0	1.5	100	99	33.3	100	99	1
	-ve	394											
**SD**	+ve	5	2	395	3	0	1.5	100	99.2	40	100	99.25	0.75
	-ve	395											
**ABON**	+ve	6	2	394	4	0	1.5	100	99	33.3	100	99	1
	-ve	394											
CLUN	+ve	37	2	363	35	0	9.3	100	91.2	5.4	100	91.25	8.75
	-ve	363											

The results using the ELISA method compared to the rapid tests showed an interesting result. For example, HBV (Table 1 and Figure 3) was identified in 8 cases using ELISA, 6 in INTEC, 2 in SD, 5 in ABON, and 6 cases in CLUN reported reactive by ELISA, compared to the false negative results: 2 in INTEC, 6 in SD, 3 in ABON, and 2 cases in CLUN not detected by the rapid test. Also, for HCV cases (Table 2 and Figure 3), there were 10 cases reported reactive by ELISA technique, only 8 cases in INTEC, 7 in SD, 7 in ABON, and 6 cases in CLUN with a positive result as compared to false negative results (2 in INTEC, 3 in SD, 3 in ABON, and 4 cases in CLUN). While in HIV (Table 3 and Figure 3), two cases were detected by ELISA and 2 cases detected by each of the four rapid tests.

## DISCUSSION

Blood transfusion is associated with many risks, especially exposure to a transfusion-transmitted infection (TTI), including HBV, HCV, and HIV [13]. In developed countries, there is a very low rate of TTIs by avoiding improper blood transfusion using regular screening for donors with highly sensitive screening such as ELISA and nucleic acid testing (NAT). This study used ELISA to compare the results with four rapid tests for detecting HBV, HCV, and HIV. The prevalence of viral markers among patients attending the Laboratories of Jiblah university hospital using the ELISA technique was 8 (2%), 10 (2.5%), and 2 (0.5%) for HBV, HCV, and HIV, respectively. In general, the seroprevalence of TTIs in our study was 20/400 (5%), which is comparable to other studies where it was identified at 2.35% and 3% [14].

The seroprevalence of HBV is similar to that of Nabehi et al. [15], who studied the prevalence of HBV in Yemen at 2.7% and 1.8% in Sana'a and Taiz, respectively. Also, the similarity of the prevalence of HBV in some Arab countries is as follows: 1.6% in Lebanon, 1.4% in Jordan, 1.3% in Morocco, 0.6% in Iraq [16], 2.4% in America, and 2.2% in Japan [17].

As for the prevalence of hepatitis C, previous studies revealed a prevalence of 2.4% in Yemen [15]. According to the World Health Organization [1], in some Arab countries, such as Saudi Arabia, the prevalence of HCV is 1–1.9%. In Australia, the prevalence rate of HCV in the adult population was 1.3% [18].

The seroprevalence of HIV in our study was 0.5% (Table 3 and Figure 3), similar to studies in Yemen but differed from other studies, where it was 1.19% in 2000 [19].

When the ELISA results were compared with the rapid tests, interesting findings were obtained for HBV, HCV, and HIV (Tables 1–3), revealing a lower sensitivity than ELISA. Furthermore, the specificity of the rapid tests was different from ELISA.

ELISA, EIA, PCR etc are some of the most sensitive tests widely used in central blood banks or well-equipped centers [20]. However, these tests and other advanced methods are time-consuming and require trained personnel. Therefore, rapid testing is used in centers with no laboratory facilities, trained workforce, or accessibility problems [21]. In addition, rapid tests are easier to use and cheaper. When used at maximum in the private sector, which serves 60% of the population, rapid testing can lead to major concerns, and its quality is sometimes at risk.

Our study showed a clear difference in the results of HBV and HCV rapid diagnostic groups in sensitivity and specificity for INTEC, SD, ABON, and CLUN, compared to the ELISA technique. The present results are similar to other studies in India and Pakistan, which showed a difference in the sensitivity and specificity of rapid test groups compared with the ELISA technique. However, our results were similar to ELISA in diagnosing HIV concerning the sensitivity of the rapid diagnostic groups but only differed in the specificity [22].

There were fewer false-negative results than false-positive results for HBV with INTEC and CLUN and fewer false-positive than false-negative results with ABON. As for HCV, the false-negative results were fewer than the false-positive with INTEC and ABON, which disagrees with another study [6]. No false-negative results were found for HIV, while 4, 3, 4, and 35 false-positive results were found with INTEC, SD, ABON, and CLUN, respectively. When screening large groups, false-positive results are preferable in many cases over false-negative, as positive serology leads to repeated testing in an alternative way to confirm the case. However, false-negative results may jeopardize the integrity of the blood.

In our study, the specificity results of the rapid test groups for HBV, HCV, and HIV were 91.2 to 99.7%. These results differ from another study [23], where the specificity obtained for HBV and HCV was 95–100%, but the sensitivity results were 86–93% for HCV and 95–98% for HBV. In this study, the sensitivity was 25–75% for HBV and 60–80% for HCV. This study showed that the SD sensitivity was lower with a ratio of 25% (true positive rates) while the specificity was higher (98.5% or higher) for false positive samples compared to Agrawal et al. [14] in detecting HBV, but the difference was not significant. Also, some poor positive results were found in HCV, which were seen in ICT with low titer positive interaction on ELISA, so it is necessary to read these rapid strips carefully while reporting results [14].

Our study concluded that these rapid test groups were not compatible with reliable diagnostic methods, so using ELISA devices for the initial screening of HBV, HCV, and HIV even in remote areas is recommended even if the cost is high.

## CONCLUSION

This study showed a significant difference in sensitivity between ELISA and rapid diagnostic immunochromatographic technique (ICT) groups.

1. For the INTEC group, the sensitivity to HBV was 75% (6 cases out of 8) and HCV 80% (8 cases out of 10), and the sensitivity rate was identical in the detection of HIV.
2. For the SD group, the sensitivity to HBV was 25% (2 cases out of 8) and HCV 70% (7 cases out of 10). The sensitivity rate was identical in the detection of HIV.
3. For the ABON group, the sensitivity to HBV was 62.5% (5 cases out of 8) and HCV 70% (7 cases out of 10). The sensitivity rate was identical in the detection of HIV.
4. For the CLUN group, the sensitivity to HBV was 75% (6 cases out of 8) and HCV 60% (6 cases out of 10). The sensitivity rate was identical in the detection of HIV.

The decrease in the sensitivity of rapid diagnostic testing proves that this method is not suitable for quality testing of infectious markers for blood donors at Jibla University Hospital. Therefore, the rapid method is not recommended when screening donors, especially for HCV and HBsAg screening.

## Personal thanks

We would like to thank all the laboratory staff of Jableh University Hospital who helped us with the practical part of this study.
